# Role of miR‐223‐3p in pulmonary arterial hypertension *via* targeting *ITGB3* in the ECM pathway

**DOI:** 10.1111/cpr.12550

**Published:** 2018-12-03

**Authors:** Aijun Liu, Yifan Liu, Bin Li, Ming Yang, Yang Liu, Junwu Su

**Affiliations:** ^1^ Department of Pediatric Cardiac Surgery Center, Beijing Anzhen Hospital Capital Medical University Beijing China; ^2^ Weifang Medicial University Weifang China

**Keywords:** *ITGB3*, MiR‐223‐3p, pulmonary arterial smooth muscle cell, pulmonary hypertension

## Abstract

**Objectives:**

To investigate the functions of miR‐223‐3p and *ITGB3* in pulmonary arterial hypertension (PAH).

**Materials and Methods:**

Microarray analysis was used to detect differentially expressed genes and microRNAs. In in vitro models, the expressions of miR‐223‐3p and *ITGB3* were detected by qRT‐PCR and Western blot. α‐SMA expression and cell proliferation were analysed by immunofluorescence and MTT assay, respectively. In in vivo models, PAH progressions were determined by measuring the levels of mPAP and RVSP. Lung and myocardial tissues were subjected to HE staining and Masson and Sirius red‐saturated carbazotic acid staining to investigate the pathological features.

**Results:**

The microarray analysis revealed that ITGB3 was upregulated, while hsa‐miR‐223‐3p was downregulated in PAH. After the induction of hypoxia, miR‐223‐3p was downregulated and *ITGB3* was upregulated in PASMCs. Hypoxia induction promoted cell proliferation and inhibited α‐SMA expression in PASMCs. Both the upregulation of miR‐223‐3p and the downregulation of ITGB3 attenuated the aberrant proliferation induced by hypoxia conditions. After approximately 4 weeks, the mPAP and RVSP levels of rats injected with MCT were decreased by the overexpression of miR‐223‐3p or the silencing of *ITGB3*. The staining results revealed that both miR‐223‐3p overexpression and *ITGB3* knockdown alleviated the pulmonary vascular remodelling and improved the PAH pathological features of rats.

**Conclusions:**

MiR‐223‐3p alleviated the progression of PAH by suppressing the expression of *ITGB3*, a finding which provides novel targets for clinical treatment.

## 1 INTRODUCTION

Pulmonary arterial hypertension (PAH) refers to a high resting mean pulmonary artery pressure (≥25 mm Hg).^1^ PAH is a progressive pulmonary vascular disease related to the dysfunction of pulmonary arterial endothelial cells (PAECs) and pulmonary arterial smooth muscle cells (PASMCs).^2^ PAH often leads to the remodelling and dysfunction of the pulmonary vasculature, right ventricular hypertrophy and even death.^3^ Optimal combination therapies are required to improve the quality of life of patients.^4^ Thus, developing novel therapeutic strategies for treating PAH is essential and urgent.

MicroRNAs (miRNAs), which are 20‐22 nucleotides in length, are endogenously expressed non‐coding RNAs that inhibit or degrade their target RNAs. MiRNAs play important roles in cell differentiation, proliferation, migration, apoptosis and stress responses.^5^ Recent studies have indicated that miRNAs can serve as biomarkers for different vascular pathologies, including early myocardial infarction and heart failure in humans.^6^ Several miRNAs have been implicated in the pathophysiological mechanisms of PAH. Sarrion et al^7^ reported that miR‐23a is correlated with pulmonary function parameters. Courboulin et al^8^ indicated that the reduction in miR‐204 levels promotes PASMC proliferation and induces resistance to apoptosis. Meloche et al^9^ reported that the downregulation of miR‐223 plays an important role in PAH, and restoring the expression of this miRNA can reverse PAH.

Integrin‐β 3 subunit gene (*ITGB3*) is known as platelet glycoprotein IIIa and antigen integrin β3 (CD61). *ITGB3* has been demonstrated to be modulated by miR‐95 to regulate the proliferation, migration and invasion of non‐small‐cell lung cancer.^10^ In addition, abnormal PASMC proliferation is a pathological feature of PAH,^11^ although there has been no specific research investigating the role of *ITGB3* in PAH. Moreover, the functions of miR‐223‐3p and *ITGB3* in PAH are still not fully understood.

In this study, we investigated the expression of miR‐223‐3p and *ITGB3* in PASMCs subjected to hypoxia in vitro and in the pulmonary artery tissues of a PAH rat model. miR‐223‐3p was found to hinder the deterioration caused by PAH, whereas *ITGB3* contributed to the progression of PAH. These discoveries suggest the great potential of miR‐223‐3p and *ITGB3* as novel PAH therapeutic targets and biomarkers for PAH.

## 2 MATERIALS AND METHODS

### 2.1 Bioinformatics analysis

The microarrays GSE33463 and GSE67597 were obtained from the Gene Expression Omnibus (GEO) database (https://www.ncbi.nlm.nih.gov/geo/). Differentially expressed genes and dysregulated pathways were uncovered through the R language and Kyoto Encyclopedia of Genes and Genomes (KEGG) database, respectively. The Linear Models for Microarray and RNA‐seq Data (Limma) package was utilized to investigate gene expression between the IPAH group and healthy controls. The threshold used to screen for upregulated and downregulated mRNA was *P* < 0.05 and |log (FC)| > 1. The differentially expressed genes were clustered using the heatmap package.

### 2.2 Cell isolation and cultivation

Healthy male Sprague Dawley (SD) rats weighing 200 g were anesthetized with 200 g/L urethane at a dose of 5 mL/kg. Pulmonary arteries were isolated in aseptic operations, and PASMCs were isolated from sections of pulmonary arteries as previously described.^12^ In the subsequent experiments, the cells were cultured at 37°C in an atmosphere with 5% CO_2_.

### 2.3 Hypoxia induction

When the cells were reached 70% confluence, a serum‐free medium was utilized to replace the initial medium that contained 10% FBS. Positive control PASMC lines were purchased from Cell Applications (San Diego, CA, USA). After being cultured for 12 hour, the cells were divided into two groups, namely the control group and the hypoxia induction group. Cells in the control group were incubated under 21% O_2_, 5% CO_2_ and balanced N_2_; cells in the hypoxia induction group were incubated under 2% O_2_, 5% CO_2_ and balanced N_2,_ as described previously.^13^

### 2.4 α‐SMA immunofluorescence

PASMCs were fixed in 4% formaldehyde for 10 minutes and then incubated in 10% goat serum for 30 minute. The cells were then incubated with the primary rabbit anti‐α‐SMA antibody (ab124964, 1:500; Abcam, Cambridge, MA, USA) overnight at 4°C. Next, the cells were incubated with the secondary antibody, DyLight 488 goat anti‐rabbit immunoglobulin G (IgG) (1:200; Beyotime, Shanghai, China), for 1 hour. 4′,6‐Diamidino‐2‐phenylindole (DAPI) was used to stain the cell nuclei (blue) at a concentration of 1.5 μmol/L. The images were observed using fluorescence microscopy.

### 2.5 Western blot

Approximately 1 × 10^7^ cells were solubilized in lysis buffer purchased from the Beyotime Institute of Biotechnology (Shanghai, China). Sodium dodecyl sulphate polyacrylamide gel electrophoresis (SDS‐PAGE) was utilized to separate the proteins. Afterwards, approximately 60 µg of the purified proteins was transferred to a polyvinylidene difluoride (PVDF) membrane. Then, the membrane with the adsorbed proteins was incubated with Tris‐buffered saline with Tween 20 (TBST) buffer obtained from Fanke Biotech Co., Ltd. (Shanghai, China) at room temperature, supplemented with 5% non‐fat milk. After 1 hour, the membrane was incubated with the primary antibodies overnight at room temperature, followed by incubation with the corresponding secondary antibody for 4 hour. In the present study, the primary antibodies used were as follows: rabbit anti‐α‐SMA (ab124964, 1:10 000 dilution; Abcam), rabbit anti‐*ITGB3* (ab218435, 1:5000 dilution), rabbit anti‐PARP‐1 (1:1000; Cell Signaling) and γH2AX (1:250; Cell Signaling). The secondary antibody was goat anti‐rabbit IgG (1:5000 dilution; Beyotime) labelled with horseradish peroxidase (HRP). An electrochemiluminescence (ECL) kit and ImageJ software from Media Cybernetics (Rockville, MD, USA) were used to determine the chemiluminescent and relative protein expression, which was presented as the density ratio compared to GAPDH.

### 2.6 Quantitative real‐time polymerase chain reaction

The RNApure total RNA extraction Kit (Bioteke Corporation, Beijing, China) was used to extract the total RNA, which was reversed transcribed using Super M‐MLV reverse transcriptase (Bioteke Corporation). To evaluate the expression levels of Rno‐miR‐223‐3p and *ITGB3*, SYBR Premix Ex Taq from TaKaRa Biotechnology (Tokyo, Japan) was utilized, and U6 and GAPDH served as the respectively internal controls. The sequences of the primers used in this study are listed in Table 1. The relative gene expression was calculated using the 2^−ΔΔCT^ method.

**Table 1:** Sequences of primers utilized in qRT‐PCR

Primers	Sequence
Rno‐miR‐223‐3p	5′‐UGUCAGUUUGUCAAAUACCCCA‐3′
U6 (Forward)	5′‐GCGCGTCGTGAAGCGTTC‐3′
U6 (Reverse)	5′‐GTGCAGGGTCCGAGGT‐3′
ITGB3 (Forward)	5′‐TCCTATGGAGACACCTGCGA‐3′
ITGB3 (Reverse)	5′‐AGGTACAGTTCACCGCGTTT‐3′
GAPDH (Forward)	5′‐TGTGAACGGATTTGGCCGTA‐3′
GAPDH (Reverse)	5′‐GATGGTGATGGGTTTCCCGT‐3′

### 2.7 Transfection

Specific siRNAs, designated *ITGB3* siRNA 1, *ITGB3* siRNA 2 and *ITGB3* siRNA 3, miR‐223‐3p mimics and inhibitor and the matched control oligonucleotides (Invitrogen, Carlsbad, CA, USA) were transfected via Lipofectamine 2000 according to the manufacturer's protocol (Invitrogen). The sequences of the oligonucleotides are listed in Table 2.

**Table 2:** Sequences of oligonucleotides utilized for transfection

Oligonucleotides	Sequence
miR‐223‐3p mimics	5′‐UGUCAGUUUGUCAAAUACCCC‐3′
Control mimics	5′‐CAUAACAACCGGCAGAAUGAGCG −3′
miR‐223‐3p inhibitor	5′‐GGGGUAUUUGACAAACUGACA‐3′
Control inhibitor	5′‐CAGUAGCATTGAGTUGCCAGAGCG‐3′
ITGB3 siRNA 1	5′‐AGGACGTGTTCACCCTGGTTCTAAA‐3′
ITGB3 siRNA 2	5′‐CAGAATCCATCGAGTTCCCAGTGAG‐3′
ITGB3 siRNA 3	5′‐GATTACCCTGTGGACATCTACTACT‐3′
Control siRNA	5′‐CAGTCTTTAGAGCTTACCTGAGCAG‐3′

### 2.8 MTT assay

3‐(4,5‐Dimethylthiazol‐2‐yl)‐2,5‐diphenyl‐tetrazolium bromide (MTT) acquired from Sigma‐Aldrich (Shanghai, China) was used to investigate cell proliferation. After 24, 48, 72 and 96 hour of culturing, 10 μL of MTT (5 mg/mL, pH 7.4) was supplemented into each well containing 1 × 10^3^ cells, which were all at the exponential growth stage. After incubation for 4 hour at 37°C, the cells were treated with 150 μL of dimethylsulphoxide (DMSO). Multiskan FC from Thermo Fisher Scientific (Waltham, MA, USA) was then applied, and the absorbance at 570 nm was measured.

### 2.9 Apoptosis assay

To investigate the rate of apoptosis, Annexin V‐FITC and propidium iodide (PI) double staining was performed. Briefly, cells were harvested, washed twice with PBS and resuspended at 1 × 10^6^ cells/mL in 100 μL of binding buffer. Next, the cells were incubated with Annexin V‐FITC and PI for 15 minute at room temperature in the dark and mixed with 400 μL of binding buffer. Flow cytometry (FACSCalibur, Becton‐Dickinson, Franklin Lakes, NJ, USA) was performed within 1 hour. The data were analysed using the Modifit Flow Cytometry Software, as previously described.^14^, ^15^

### 2.10 Dual luciferase reporter assay

Recombinant luciferase reporter vectors containing the *ITGB3* 3′‐untranslated region (3′UTR) wild type (WT) and mutant type (MUT) were constructed. MiR‐223‐3p mimics or the negative control was co‐transfected with ITGB3 MUT and ITGB3 WT into human foetal kidney 293 T cells (HEK‐293 T) using Lipofectamine 2000 (Invitrogen). Dual luciferase measurements were performed using a Glomax 20/20 Luminometer (YuanPingHao Biotechnology Co Ltd, Beijing, China) 48 hour after the HEK‐293 T were transfected.

### 2.11 Animal model

Rats were divided into the experimental group and the control group, which were respectively treated with 1% monocrotaline (MCT, pH 7.4, 60 mg/kg) and 0.9% normal saline via intraperitoneal injection. After approximately 4 weeks, severe PAH was observed in the experimental group.^16^

To determine the effects of *ITGB3* and miR‐223‐3p in vivo, pGenesil‐1‐sh‐*ITGB3* was constructed. The expression promoter of control plasmid pGenesil‐1‐HK U6 as well as short hairpin‐*ITGB3* (sh‐*ITGB3)* was cloned into the adenovirus shuttle vector pAdTrack to construct the recombinant adenovirus shuttle plasmids pAdTrack‐U6‐sh‐*ITGB3* and pAdTrack‐U6‐HK. Additionally, agomiR‐223‐3p and control agomir were also constructed based on the sequences shown in Table 2. Rats with established PAH were nebulized with agomiR‐223‐3p (20 µmol/L once per week for 2 weeks) or adenovirus (1.5 × 10^8^ pfu/L, 50 µL once per week for 2 weeks).^17^, ^18^

### 2.12 Haemodynamics

The pulmonary pressure changes were measured. In brief, a 13‐cm‐long, heparin‐priming polyethylene catheter (outer diameter, 0.9 mm) connected to PowerLab 16/30 (ADInstruments, Dunedin, New Zealand) through a pressure transducer was introduced into the right external jugular vein and advanced into the right ventricle and the main pulmonary artery. The right ventricular systolic pressure (RVSP), pulmonary arterial systolic pressure (PASP), total pulmonary resistance (TPR) and mean pulmonary arterial pressure (mPAP) were recorded. Meanwhile, the cardiac output (CO) and end‐diastolic pressure were assessed, as previously described.^19^, ^20^, ^21^, ^22^

### 2.13 Haematoxylin‐eosin (HE) staining

Paraffin‐embedded tissue sections were roasted and dried for 20 minute in the oven at 75°C, followed by immersion in xylene for 15 minute, anhydrous ethanol for 5 minute, 95% ethanol for 5 minute, 80% ethanol for 5 minute and 70% ethanol for 5 minute. All operations were conducted twice. Briefly, after the sections were dried, haematoxylin was used to stain the nuclei, and eosin was used to stain the cytoplasm after dewaxing and hydration, as previously described.^23^, ^24^ To examine the degree of fibrosis, microscopic images of the HE‐stained tissues were graded according to the Ashcroft method.^25^

### 2.14 Masson staining

For Masson staining, paraffin‐embedded tissue sections were stained with haematoxylin for 1 minute and then Masson Li Chunhong acid fuchsine solution for 5 minute. The detailed histological protocols were previously described.^26^

### 2.15 Sirius red‐saturated carbazotic acid staining

Paraffin‐embedded tissue sections were washed with distilled water and then stained with celestine blue for 10 minute followed by three washes with distilled water. Then, Sirius red‐saturated carbazotic acid was applied for 20 minute. Afterwards, the sections were treated with 0.2% ethylic acid for 1 minute, twice. After dehydration, transparency and sealing, the tissues were observed using ordinary optical or polarized light microscopy.^27^, ^28^

### 2.16 Immunohistochemistry

Immunohistochemistry (IHC) analysis was performed on 5‐μm paraffin‐embedded tissue sections that were rehydrated in a graded series of ethanol (99%‐70%) and finally in distilled water. For *ITGB3* staining, blocking of non‐specific antibody binding was blocked with 3% bovine serum albumin (BSA) in Tris‐buffered saline supplemented with 0.05% Tween 20 (TBST). A primary antibody against rabbit *ITGB3* (ab218435, 1:100; Abcam) was diluted in 1% BSA in TBST. The antigen‐antibody complex was visualized using a horseradish peroxidase‐conjugated IgG secondary antibody (1:200; Beyotime) followed by 3,3′‐diaminobenzidine (DAB). ITGB3 immunostaining was scored as follows: score 0, no expression; score 1, weak expression; score 2, moderate expression; score 3, strong expression.^29^

### 2.17 Data analysis

Data were presented as the mean ± standard deviation (SD) of at least three independent replicates. Data were analysed with GraphPad Prism 6.0 (GraphPad Software, La Jolla, CA, USA), and comparisons between two groups were analysed by the Student's *t* test. Multiple groups were compared with one‐way ANOVA. Differences were considered significant at values of *P* < 0.05.

## 3 RESULTS

### 3.1 Downregulation of miR‐223‐3p in IPAH and related dysregulated pathways

**Figure 1:**
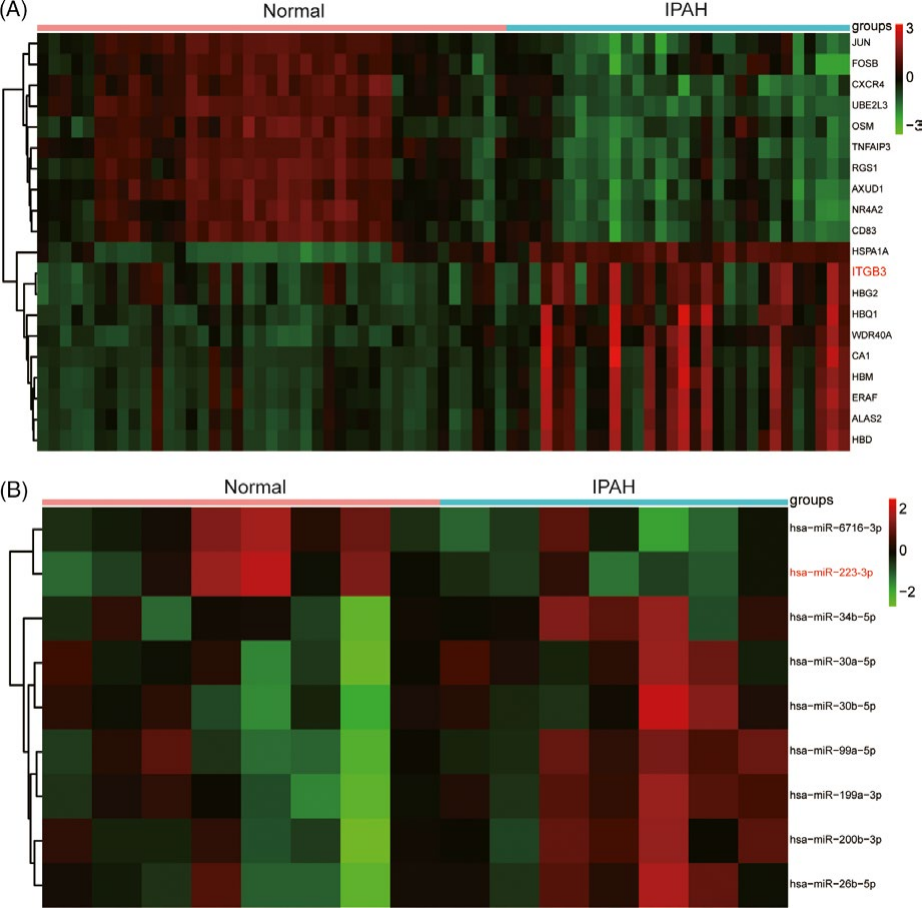
MiR‐223‐3p and *ITGB3* are differentially expressed in IPAH patients. A, According to GSE33463, differentially expressed genes included *ITGB3* in IPAH. B, After analysing the data of GSE67597, differentially expressed miRNAs were detected, including miR‐223‐3p in IPAH

**Figure 2:**
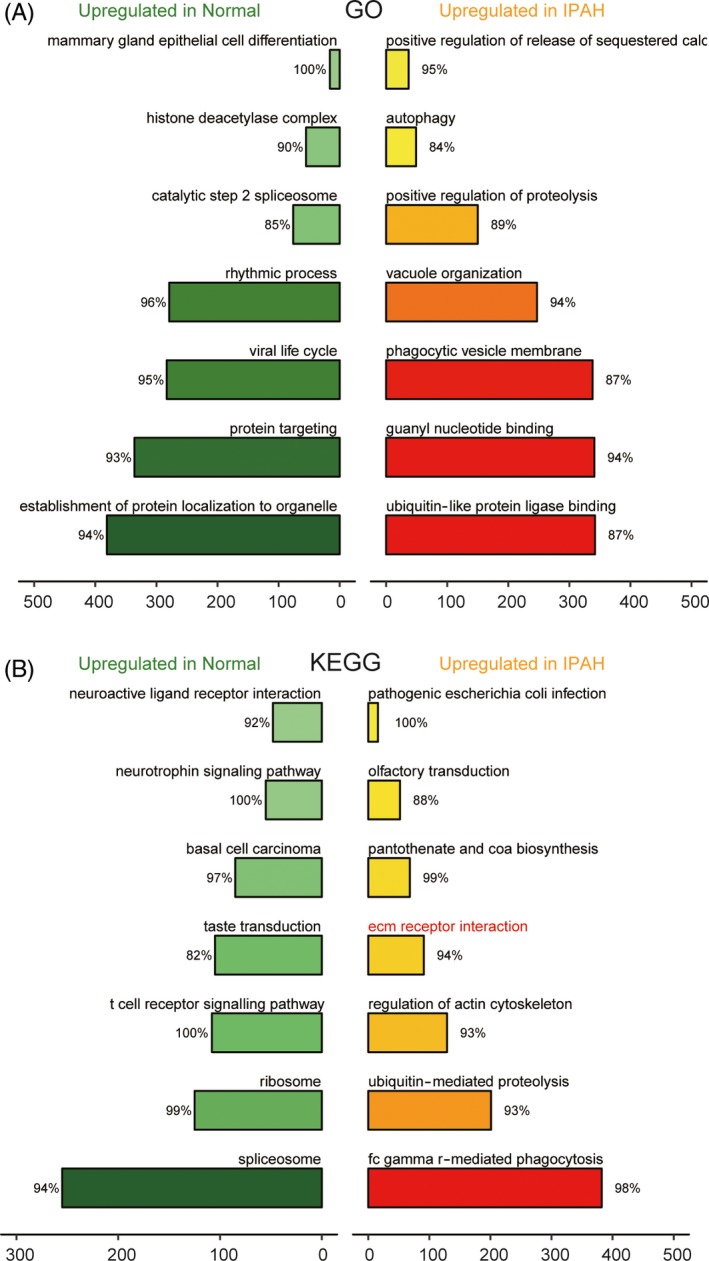
Gene oncology (GO) and Kyoto encyclopedia of genes and genomes (KEGG) pathway analysis of differentially expressed genes in IPAH. A, The seven most distinctively activated GO pathways between healthy and IPAH tissues. B, The seven most distinctively activated KEGG pathways in healthy and IPAH tissues

**Figure 3:**
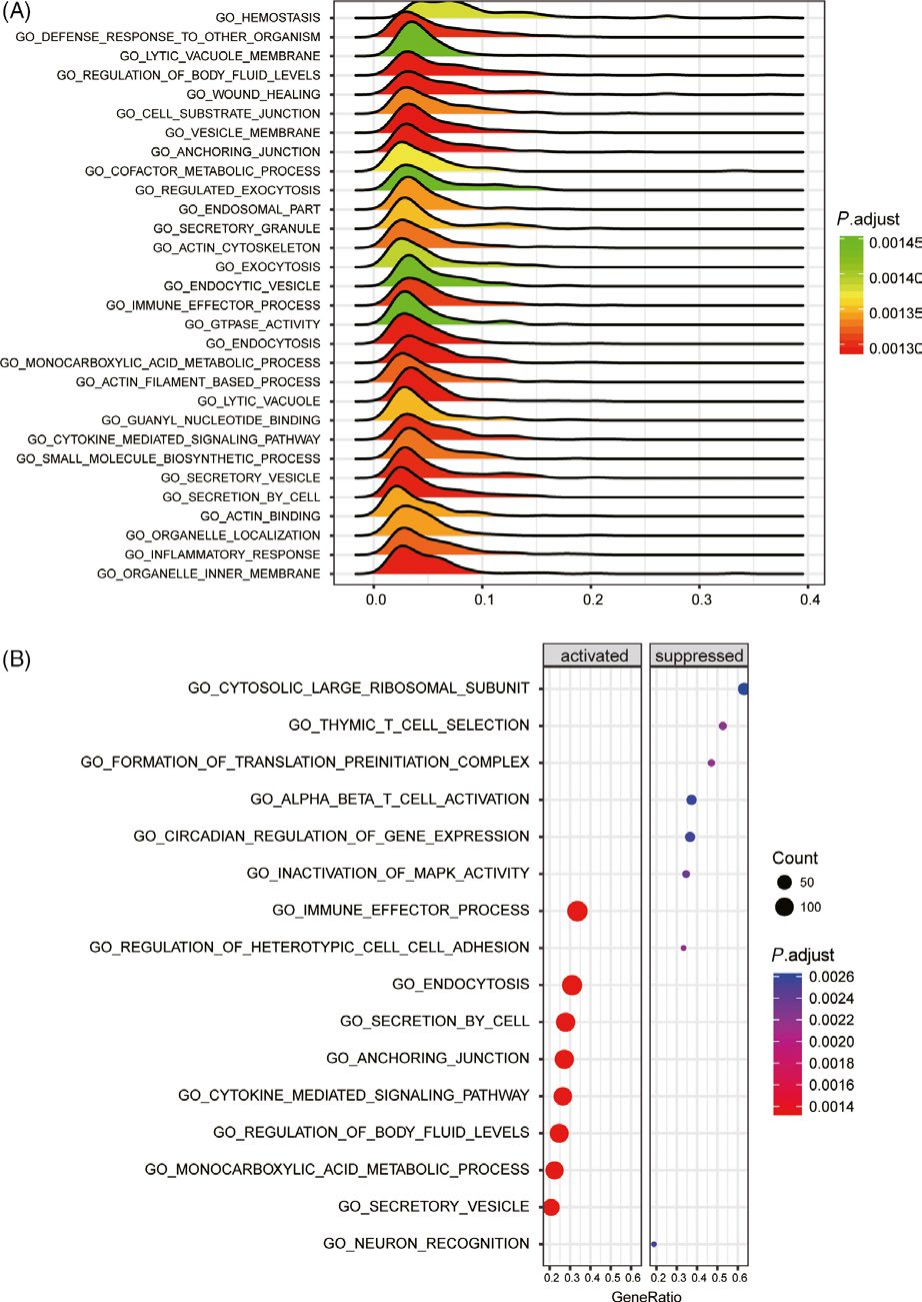
Results of gene oncology (GO) analysis. A and B, The GO analysis revealed differentially modulated pathways in the IPAH group compared to the normal group. In the dotplot, the graph size represents the number of genes. The colour represents the *P* value. In the joyplot, the enrichment significance (the adjusted *P* value) is reflected by the colour intensity of the peaks. Pathways with ridges on the left side of 0 were downregulated, while those with ridges on the right side of 9 were upregulated. The gene ratio in the horizontal axis represents the proportion of differential genes in the gene set

**Figure 4:**
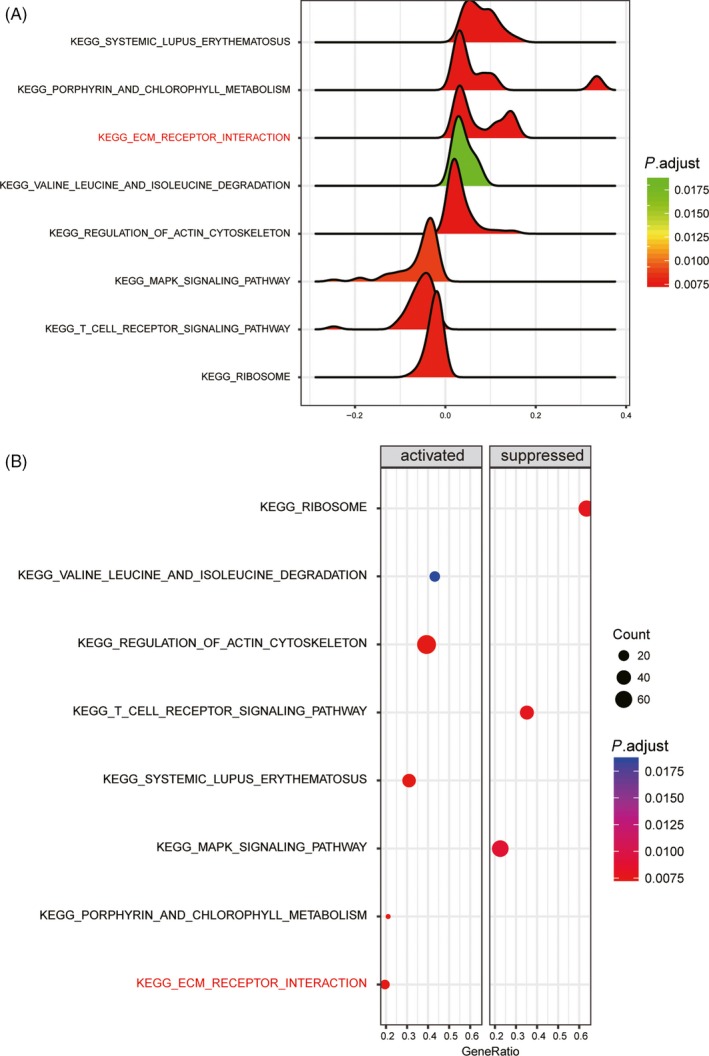
Results of Kyoto encyclopedia of genes and genomes (KEGG) analysis. A and B, The KEGG analysis revealed differentially modulated pathways in the IPAH group compared to the normal group, including the extracellular matrix (ECM) receptor interaction pathway. In the dotplot, the graph size represents the number of genes. The colour represents the *P* value. In the joyplot, the enrichment significance (the adjusted *P* value) is reflected by the colour intensity of the peaks. Pathways with ridges on the left side of 0 were downregulated, while those with ridges on the right side of 9 were upregulated. The gene ratio in the horizontal axis represents the proportion of differential genes in the gene set

Differentially expressed genes and dysregulated pathways were determined using the R language and KEGG database, respectively. Figure 1A shows the top 10 differentially expressed genes with aberrantly high and low expression. ITGB3 was shown to be upregulated in IPAH tissues. As shown in Figure 1B, hsa‐miR‐223‐3p was downregulated in patients with IPAH. The top 14 significantly activated and inactivated pathways in IPAH are shown in Figure 2A. The Gene Oncology (GO) analysis revealed that these co‐expressed genes in IPAH were associated with several biological processes, including autophagy and phagocytic vesicle membrane (Figure 2A). The KEGG analysis results revealed upregulated pathways in IPAH, such as olfactory transduction, pantothenate and CoA biosynthesis, regulation of actin cytoskeleton and ubiquitin‐like protein ligase binding. Compared to the normal group, the extracellular matrix (ECM) receptor interaction pathway was significantly upregulated in the IPAH group (Figure 2B). The joyplot (Figure 3A) and the dotplot (Figure 3B) illustrate the activated and suppressed GO pathways in IPAH tissues. Figure 4A,B illustrates the activated and suppressed KEGG pathways in IPAH tissues, including the ECM receptor interaction pathway.

### 3.2 Visual characterization of PASMC and analysis of the expressions of miR‐223‐3p and *ITGB3* after hypoxia induction

**Figure 5:**
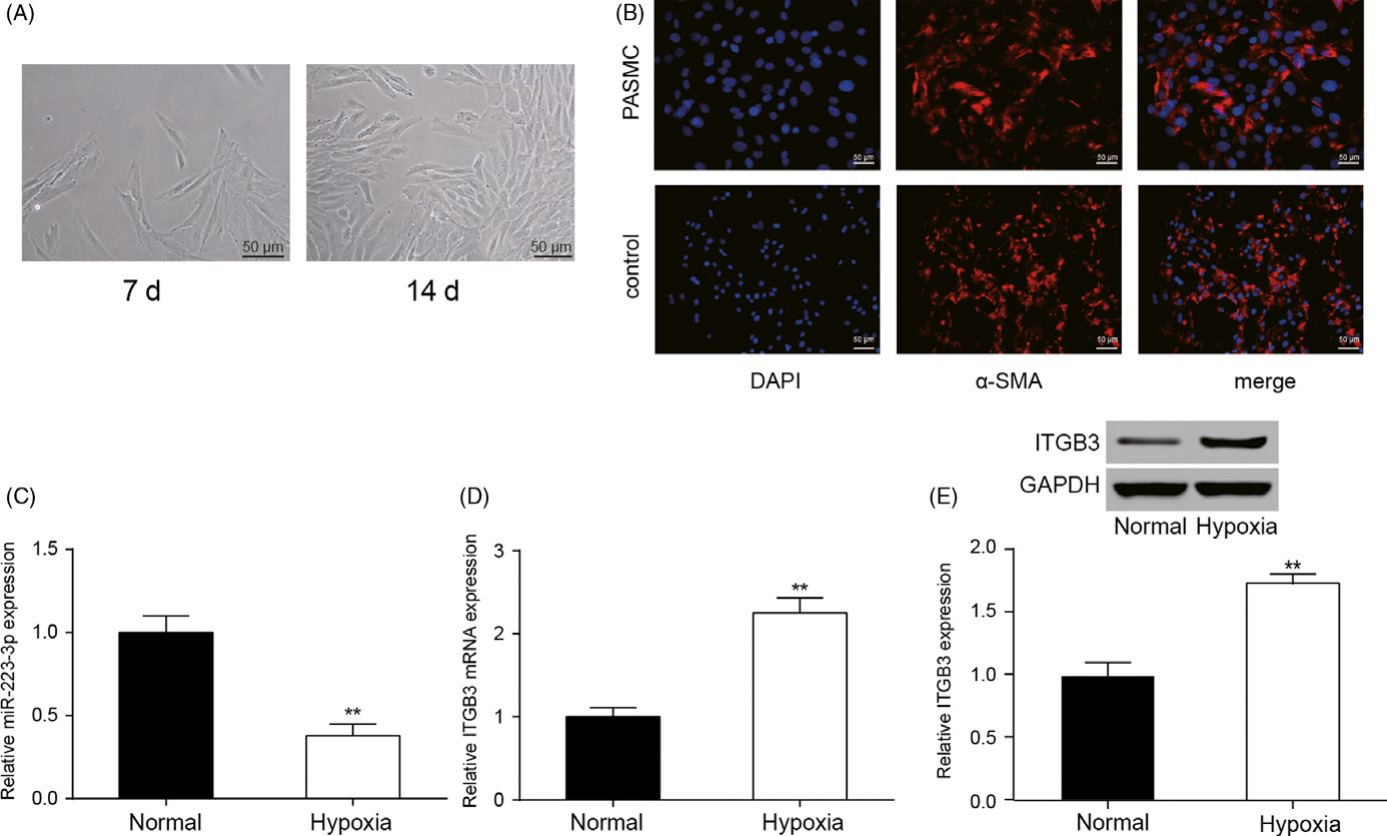
Characterization of pulmonary arterial smooth muscle cells (PASMCs) and analysis of the expressions of miR‐223‐3p and *ITGB3* the induction of after hypoxia. A, Photographs of PASMC were screened, magnification, ×200; scale bar: 50 µm. B, PASMCs were characterized by α‐smooth muscle actin (α‐SMA) immunofluorescence, magnification, ×400; scale bar: 50 µm. C and D, QRT‐PCR was utilized to evaluate the expression levels of miR‐223‐3p and *ITGB3* in the hypoxic cells compared with the normal cells. E, *ITGB3* protein expression was determined in normal and hypoxic cells by Western blot. All experiments were performed in triplicate. **P < 0.01

PASMCs were observed under the microscope and “peak and valley” characteristics were exhibited (Figure 5A). α‐SMA was strongly expressed in PASMCs (Figure 5B). After the induction of hypoxia, the expression of miR‐223‐3p was suppressed in the hypoxia group (Figure 5C, *P *< 0.01), whereas *ITGB3* expression was elevated in the hypoxia group compared with normal group (Figure 5D,E, *P *< 0.01).

### 3.3 MiR‐223‐3p and *ITGB3* expression levels after transfection

**Figure 6:**
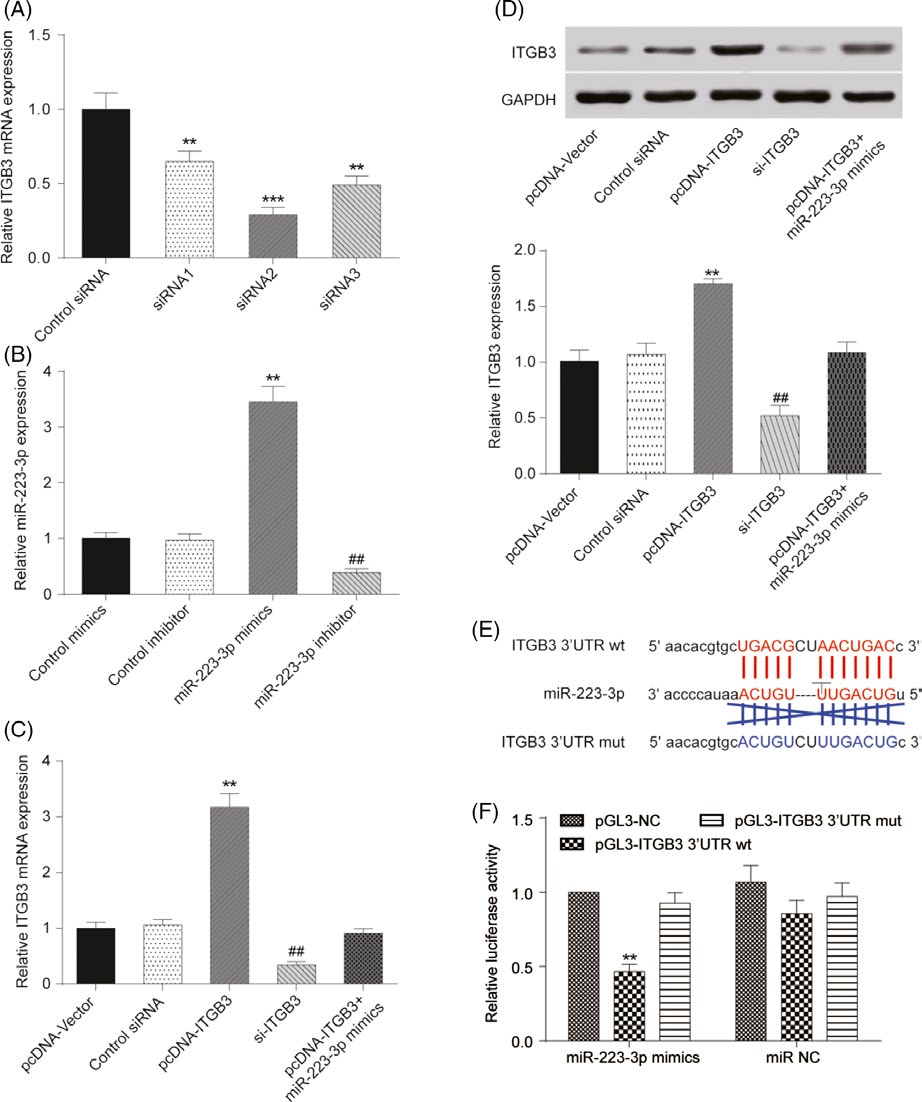
MiR‐223‐3p and *ITGB3* expression level after transfection. A, *ITGB3* expression after transfection with three specific siRNAs was determined. B, MiR‐223‐3p expression level after transfection with miR‐223‐3p mimics or inhibitor was confirmed via qRT‐PCR. C, The *ITGB3* mRNA level after transfection with pcDNA‐*ITGB3* or si‐*ITGB3* transfection was compared with matched control groups via qRT‐PCR. D, The protein expression of *ITGB3* was measured and compared with the matched control group after transfection with pcDNA‐*ITGB3* and si‐*ITGB3* or co‐transfection with pcDNA‐*ITGB3* and miR‐223‐3p mimics. E, Design of ITGB3 3′UTR (wild type and mutant) was the target sequence. F, The dual luciferase reporter assay was performed, and decreased luciferase activity was observed in miR‐223‐3p mimics + pGL3‐ITGB3 wt group. All experiments were performed in triplicate. **P < 0.01 compared with control siRNA, control mimics, pcDNA Vector or pGL3‐NC group; ^##^ *P *< 0.01 compared with control inhibitor or control siRNA

**Figure 7:**
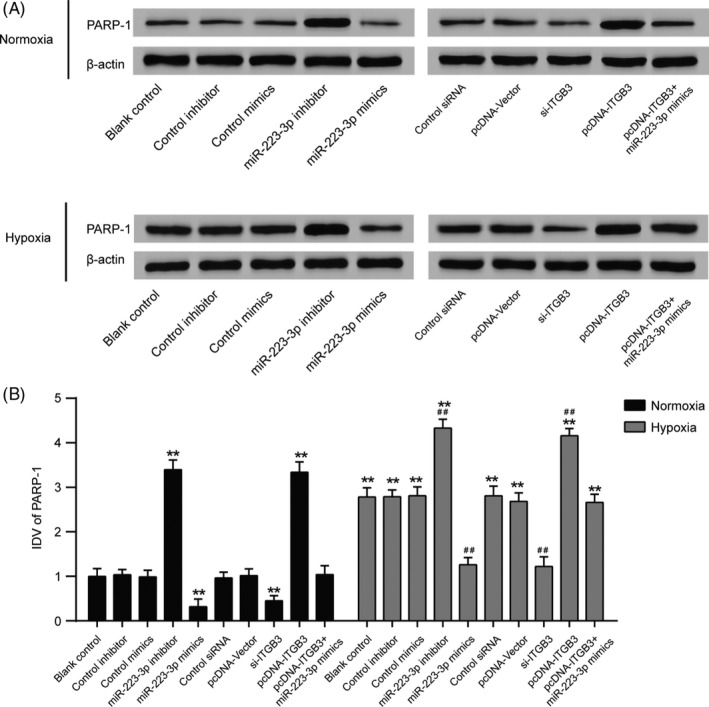
Poly [ADP‐ribose] polymerase 1 (PARP‐1) expression level in pulmonary arterial smooth muscle cells (PASMCs) cultured under normal or hypoxic conditions after transfection. Other targets of miR‐223 were measured, including PARP‐1. A, After being cultured in either normoxic or hypoxic conditions for 36 h, Western blot analysis was performed to measure the protein level in all groups. B, Relative integrated density value (IDV) analysis of PARP‐1. All experiments were performed in triplicate. **P < 0.01 compared with the blank control in the normoxia treatment group; ^##^ *P *< 0.01 compared with blank control in the hypoxia group

**Figure 8:**
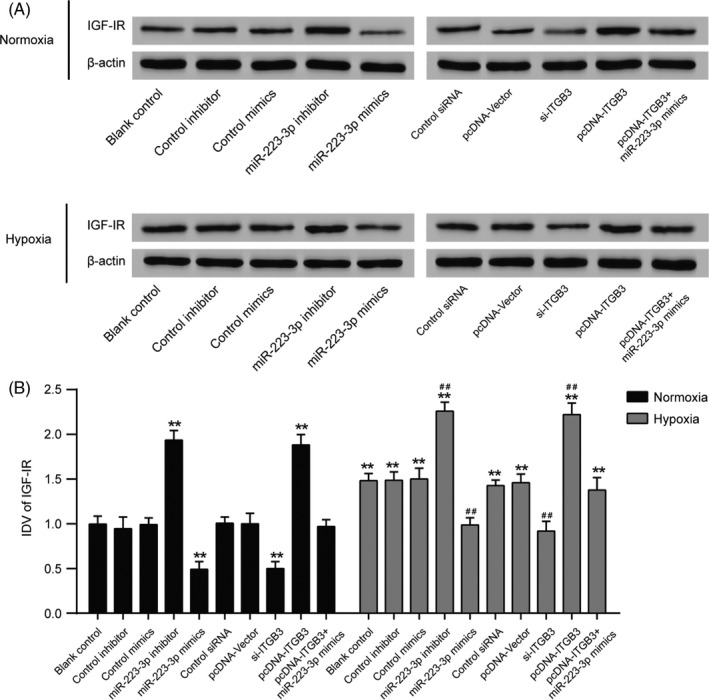
Insulin‐like growth factor‐I receptor (IGF‐IR) expression in pulmonary arterial smooth muscle cells (PASMCs) cultured under normal or hypoxic conditions after transfection. Other targets of miR‐223 were measured, including IGF‐IR A, After being cultured in either normoxic or hypoxic conditions for 36 h, Western blot analysis was performed to measure the protein levels in all groups. B, Relative integrated density value (IDV) analysis of IGF‐IR All experiments were performed in triplicate. **P < 0.01 compared with the blank control in the normoxia treatment group; ^##^ *P *< 0.01 compared with the blank control in the hypoxia group

Three different specific siRNAs against *ITGB3* were transfected into PASMCs. SiRNA2 exhibited the highest interference efficiency (Figure 6A, *P *< 0.001) and was therefore selected for use in further experiments and designated as si‐*ITGB3*. miR‐223‐3p expression in PASMCs was significantly elevated after transfection with miR‐223‐3p mimics, whereas transfection with miR‐223‐3p inhibitor had the opposite effect (Figure 6B, *P *< 0.01). Transfection with pcDNA‐*ITGB3* resulted in the upregulated mRNA expression of *ITGB3* compared with the control plasmid, whereas in the si‐*ITGB3* group, the mRNA expression of *ITGB3* was significantly attenuated (Figure 6C, *P *< 0.01). In addition, the Western blot analysis confirmed these results (Figure 6D). The luciferase reporting assay using the ITGB3 wild‐type 3′UTR and mutant 3′UTR confirmed the direct target relationship between miR‐223‐3p and the ITGB3 3′UTR (Figure 6E,F). In addition, Jolyane Meloche et al^9^ reported that the downregulation of miR‐223 promotes the expression of poly [ADP‐ribose] polymerase 1(PARP‐1) in human PAH‐PASMC. Shi et al^30^ reported that the downregulation of miR‐223 in the lung was associated with an increased expression of the miR‐223 target, insulin‐like growth factor‐I receptor (IGF‐IR) and insulin‐like growth factor‐l (IGF‐1) downstream signalling. To confirm our findings, PARP‐1 and IGF‐IR levels were detected by Western blot. As shown in Figure 7A,B and 8A,B, after the induction of hypoxia, PARP‐1 and IGF‐IR expression increased significantly. Notably, both the suppression of ITGB3 and the elevated expression of miR‐223‐3p impaired the hypoxia‐induced expression of PARP‐1 and IGF‐IR Moreover, the overexpression of miR‐223‐3p attenuated the impact of pcDNA‐ITGB3 in both the normoxia and hypoxia group.

### 3.4 The proliferation of PASMCs after transfection under normal or hypoxic conditions

**Figure 9:**
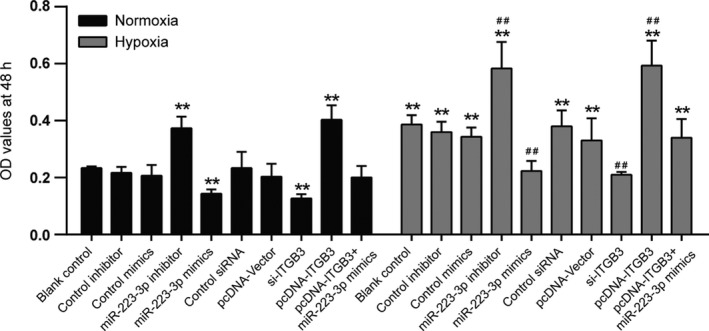
The proliferation of pulmonary arterial smooth muscle cell (PASMC) after transfection under normal or hypoxic conditions. Cell proliferation of PASMCs was measured, after transfection with miR‐223‐3p mimics or miR‐223‐3p inhibitor or si‐*ITGB3* or pcDNA‐*ITGB3* at 48 h. All experiments were performed in triplicate. **P < 0.01 compared with the blank control in the normoxia treatment group; ^##^ *P *< 0.01 compared with the blank control in hypoxia group

Cells transfected with all methods exhibited a strong proliferative ability under hypoxic conditions (Figure 9, *P* < 0.01). The inhibition of miR‐223‐3p or overexpression of ITGB3 efficiently increased the optical density (OD) values and facilitated cell proliferation under both normoxic and hypoxic conditions. Meanwhile, an elevated expression of miR‐223‐3p reverses the effect of hypoxia compared with blank control group. Furthermore, upregulated expression of miR‐223‐3p also attenuated the positive effect of ITGB3 overexpression under both normoxic and hypoxic conditions.

### 3.5 The rate of apoptosis and the expression of γ‐H2AX expression level in PASMCs after transfection under normal or hypoxic conditions

**Figure 10:**
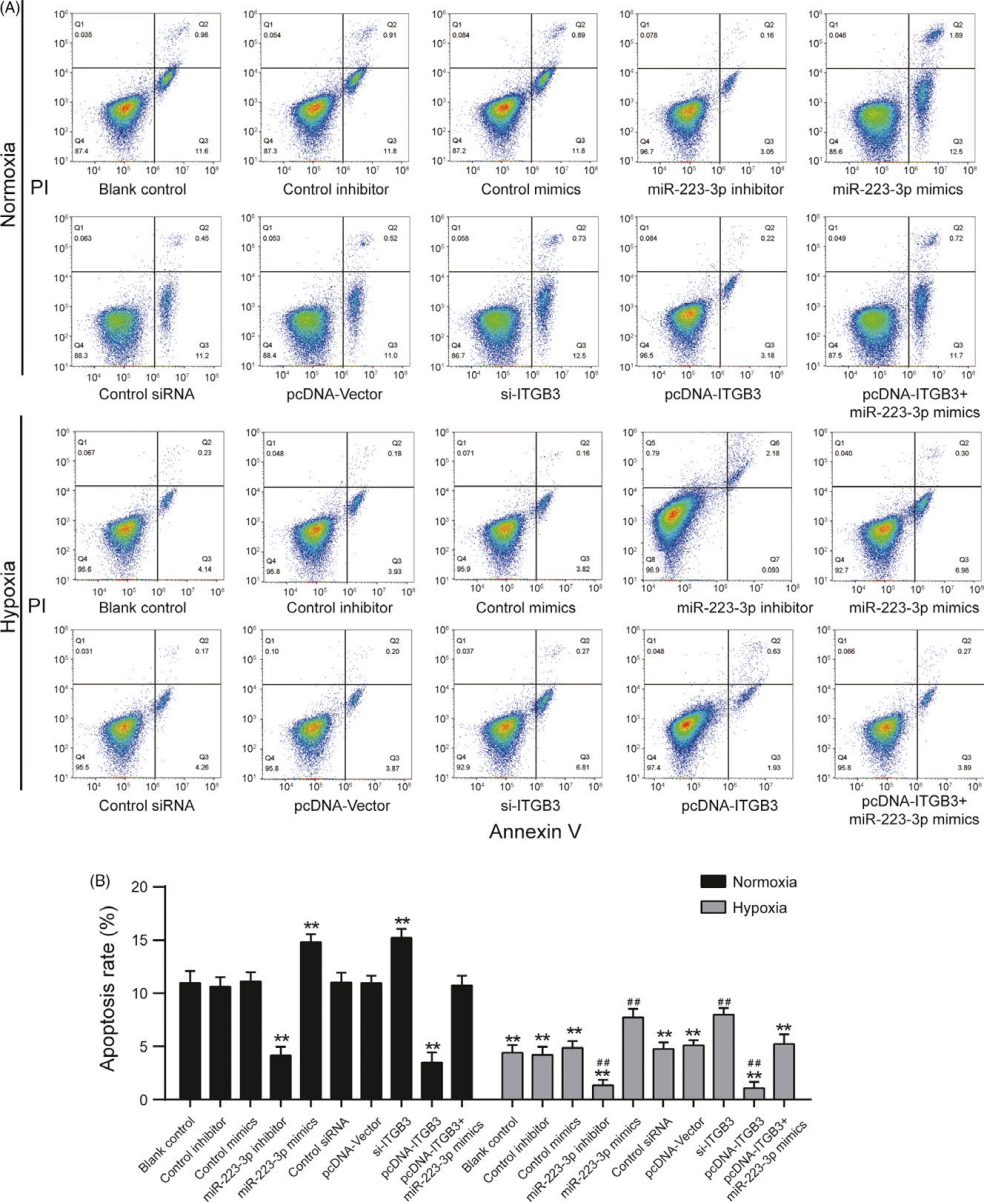
miR‐223 regulates the apoptosis of pulmonary arterial smooth muscle cell (PASMCs) after transfection under normal or hypoxic condition. Flow cytometric analysis was used to evaluate the effects of miR‐223‐3p mimics or miR‐223‐3p inhibitor or si‐*ITGB3* or pcDNA‐*ITGB3* transfection at 48 h on apoptosis of PASMCs. A, The *x*‐axis represents Annexin V‐FITC staining, and *y*‐axis represents PI staining. Quadrant Q4 is Annexin V‐FITC‐and PI negative, representing viable cells. Quadrant Q3 is Annexin V‐FITC positive and PI negative, representing cells in early‐stage apoptosis viable cells. Quadrant Q1 is Annexin V‐FITC negative and PI positive, representing dead cells. Quadrant Q2 is Annexin V‐FITC and PI positive, representing cells in late‐stage apoptosis. B, The percentage of early‐ and late‐stage apoptotic cells is represented in the histogram. Data were representative of three independent experiments. **P < 0.01 compared with the blank control in the normoxia treatment group; ^##^ *P *< 0.01 compared with the blank control in the hypoxia group

**Figure 11:**
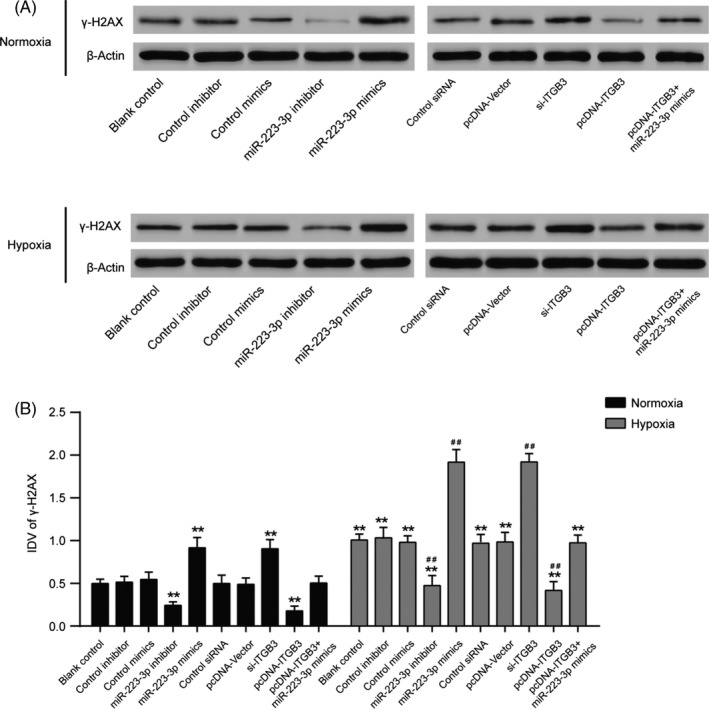
The expression of γ‐H2AX in pulmonary arterial smooth muscle cell (PASMCs) cultured under normal or hypoxic conditions after transfection. The DNA damage sensor γ‐H2AX is expressed at different levels in each group. A, After being cultured in either normoxic or hypoxic conditions for 36 h, a Western blot assay was performed to measure the protein level in all groups. B, Relative integrated density value (IDV) analysis of γ‐H2AX. Data were representative of three independent experiments. **P < 0.01 compared with the blank control in the normoxia treatment group; ^##^ *P *< 0.01 compared with the blank control in the hypoxia group

Cells transfected with all methods exhibited a low rate of apoptosis under hypoxic conditions compared to normoxic conditions (Figure 10A,B, *P* < 0.01). The upregulation of miR‐223‐3p promoted apoptosis as did the downregulation of ITGB3, whereas the inhibition of miR‐223‐3p or the overexpression of ITGB3 markedly decreased the apoptosis rate under both normoxic and hypoxic conditions. Further, the upregulated expression of miR‐223‐3p attenuated the positive effect of ITGB3 overexpression under both normoxic and hypoxic conditions. In addition, to confirm whether miR‐223 modulation affects apoptosis through DNA damage, the expression of DNA damage sensor γ‐H2AX^31^ was detected. As shown in Figure 11A,B, after the induction of hypoxia, γ‐H2AX expression increased significantly, indicating that, indeed, miR‐223 modulation affects cells apoptosis through DNA damage. Suppression of ITGB3 or elevated expression of miR‐223‐3p enhanced the expression of γ‐H2AX induced by hypoxia (Figure 11A,B, *P* < 0.01).

### 3.6 The expression of α‐SMA in PASMCs cultured under normal or hypoxic condition after transfection

**Figure 12:**
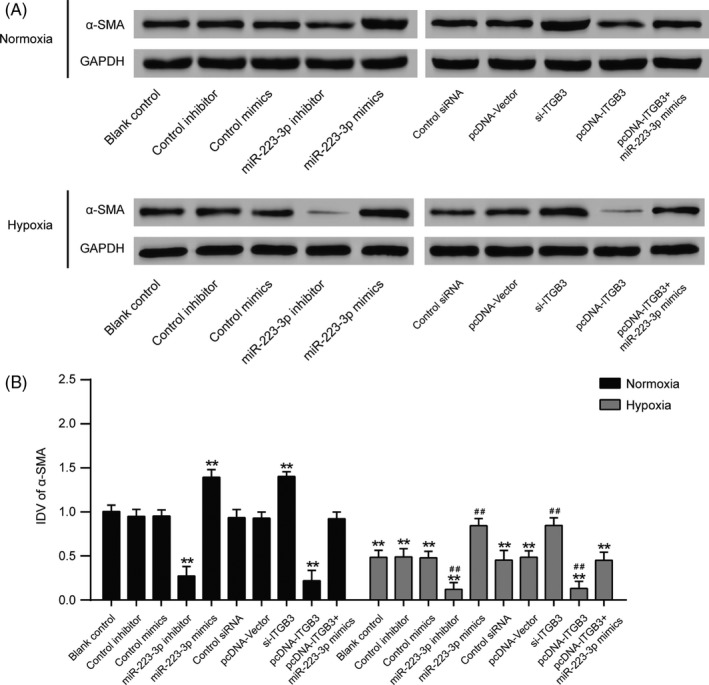
The expression of α‐SMA in pulmonary arterial smooth muscle cell (PASMCs) cultured under normal or hypoxic conditions after transfection. A, After being cultured in either normoxic or hypoxic conditions for 36 h, a Western blot assay was performed to measure the protein levels in all groups. B, Relative integrated density value (IDV) analysis of α‐SMA. All experiments were performed in triplicate. **P < 0.01 compared with the blank control in the normoxia treatment group; ^##^ *P *< 0.01 compared with the blank control in the hypoxia group

Transfected cells were incubated under normal or hypoxic conditions, and then, the total proteins were extracted. Because the phenotypic modulation of pulmonary artery smooth muscle cells (PASMCs) can be triggered by hypoxia,^32^, ^33^ and α smooth muscle actin (α‐SMA), a phenotype marker of PASMCs, is the first known protein to be expressed during the differentiation of the smooth muscle cell (SMC) in development,^34^ Western blot was employed to evaluate the protein expression of α‐SMA. As shown in Figure 12A,B, after cells were incubated in a hypoxic environment, α‐SMA expression decreased significantly, indicating a phenotypic change. Importantly, both the suppression of ITGB3 and the elevated expression of miR‐223‐3p promoted the expression of α‐SMA induced by hypoxia. Moreover, overexpression of miR‐223‐3p attenuated the impact of pcDNA‐ITGB3 in both the normoxia and hypoxia group.

### 3.7 Rat model building

The lung tissues of the control rats presented were pink with smooth and full surfaces. The rats in the MCT group exhibited clearly reduced activity levels, reduced consumption of food, disordered hair, and cyanotic noses and lips after 2 weeks. The chests were analysed after 4 weeks. The lung tissue was grey with poor elasticity. The right ventricular wall was found to be thickened after the heart was cut. Notably, the agomiR‐223‐3p and sh‐*ITGB3* adenovirus transfection groups exhibited higher activity levels and a few bruises at lung tissue surface. However, a situation similar to the control group was observed for the agomiR‐223‐3p and sh‐*ITGB3* co‐transfection groups. The observed phenotypes of each group are presented in Table 3. After 4 weeks, weight loss was observed in the MCT group compared with the other group, and the co‐transfection group was healthier overall compared with the sh‐*ITGB3* adenovirus transfection group. Moreover, the weight of the rats in the co‐transfection group was close to that of the rats in the control group. The weight of each group is presented in Table 4.

**Table 3:** Phenotypes observed per group

	Normal	MCT	MCT+ agomiR‐223‐3p	MCT+ sh‐ITGB3	MCT + agomiR‐223‐3p + sh‐ITGB3	MCT + NC
Lung tissues	Pink, smooth and full surface	Gray, poor elasticity, bruises	A few bruises	A few bruises	A fewer bruises	Bruises
Hair	More orderly	Disordered	Less order	Less order	Ordered	Disordered
Nose and lips	Pink	Cyanotic	Mauve	Mauve	Almost pink	Cyanotic
Right ventricular wall	Thin	Thickest	Thicker	Thicker	Thick	Thickest

**Table 4:** Weight of rats in indicated groups

Group	Baseline (g)	Week 2 (g)	Week 4 (g)
Normal	224.41 ± 7.92	241.85 ± 10.26	266.23 ± 13.81
MCT	228.14 ± 8.44	237.71 ± 7.11	239.34 ± 10.13*
MCT + agomiR‐223‐3p	222.18 ± 6.67	239.17 ± 8.50	256.18 ± 7.75^#^
MCT + sh‐ITGB3	225.52 ± 7.05	239.88 ± 8.61	259.77 ± 9.03^#^
MCT + agomiR‐223‐3p + sh‐ITGB3	226.12 ± 6.25	236.18 ± 9.20	262.71 ± 10.11^#^
MCT + NC	227.31 ± 8.01	235.78 ± 6.76	241.62 ± 8.16

*P < 0.05 compared with week 4 of normal group; ^#^P < 0.05 compared with week 4 of MCT+NC group.

### 3.8 Hemodynamic and the index of right ventricular hypertrophy

**Figure 13:**
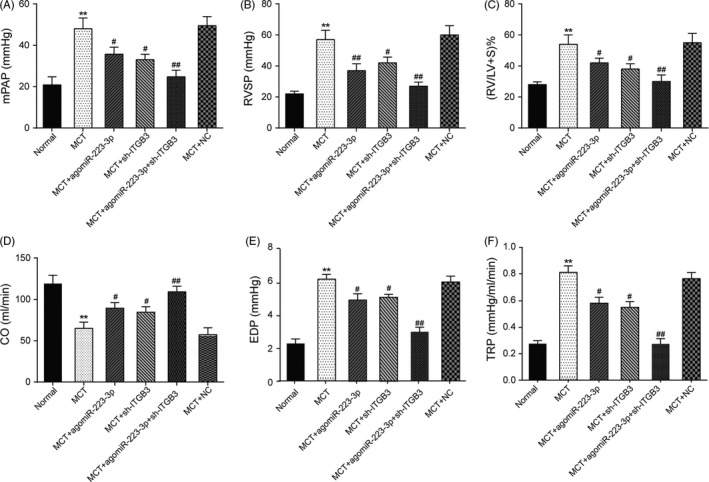
Haemodynamics and the index of right ventricular hypertrophy. A, mean pulmonary arterial pressure (mPAP) levels of each group after 4 wk. B, right ventricular systolic pressure (RVSP) levels of each group after 4 wk. C, Right ventricular mass ratio of each group after 4 wk. D, CO of each group. E, EDP of each group. F, TRP of each group, n = 8 for each group; all experiments were performed in triplicate. **P < 0.01 compared with the normal group; ^#^ *P *< 0.05, ^##^ *P *< 0.01 compared with the MCT group rats

The levels of mPAP, TRP, EDP and RVSP in the MCT induced rats were elevated compared with the normal rats at 4 weeks, and the right ventricular mass ratio of the rats in the MCT group was also increased (*P *< 0.01) with obvious pulmonary hypertension. In addition, the CO of the experimental rats was attenuated compared with the normal rats, but the overexpression of miR‐223‐3p and the knockdown of *ITGB3* increased the CO compared with the MCT group. The levels of mPAP and RVSP in the rats with overexpressed miR‐223‐3p and downregulated *ITGB3* expression were significantly elevated compared with normal control, but the situation was improved obviously compared with MCT group with a lower right ventricular mass ratio (*P *< 0.05). Moreover, the levels of TRP and EDP in the rats with overexpressed miR‐223‐3p and downregulated *ITGB3* expression were significantly elevated, whereas the CO of this group was significantly decreased compared with the normal controls (Figure 13A‐F, *P *< 0.01).

### 3.9 MiR‐223‐3p and ITGB3 expression in the pulmonary artery

**Figure 14:**
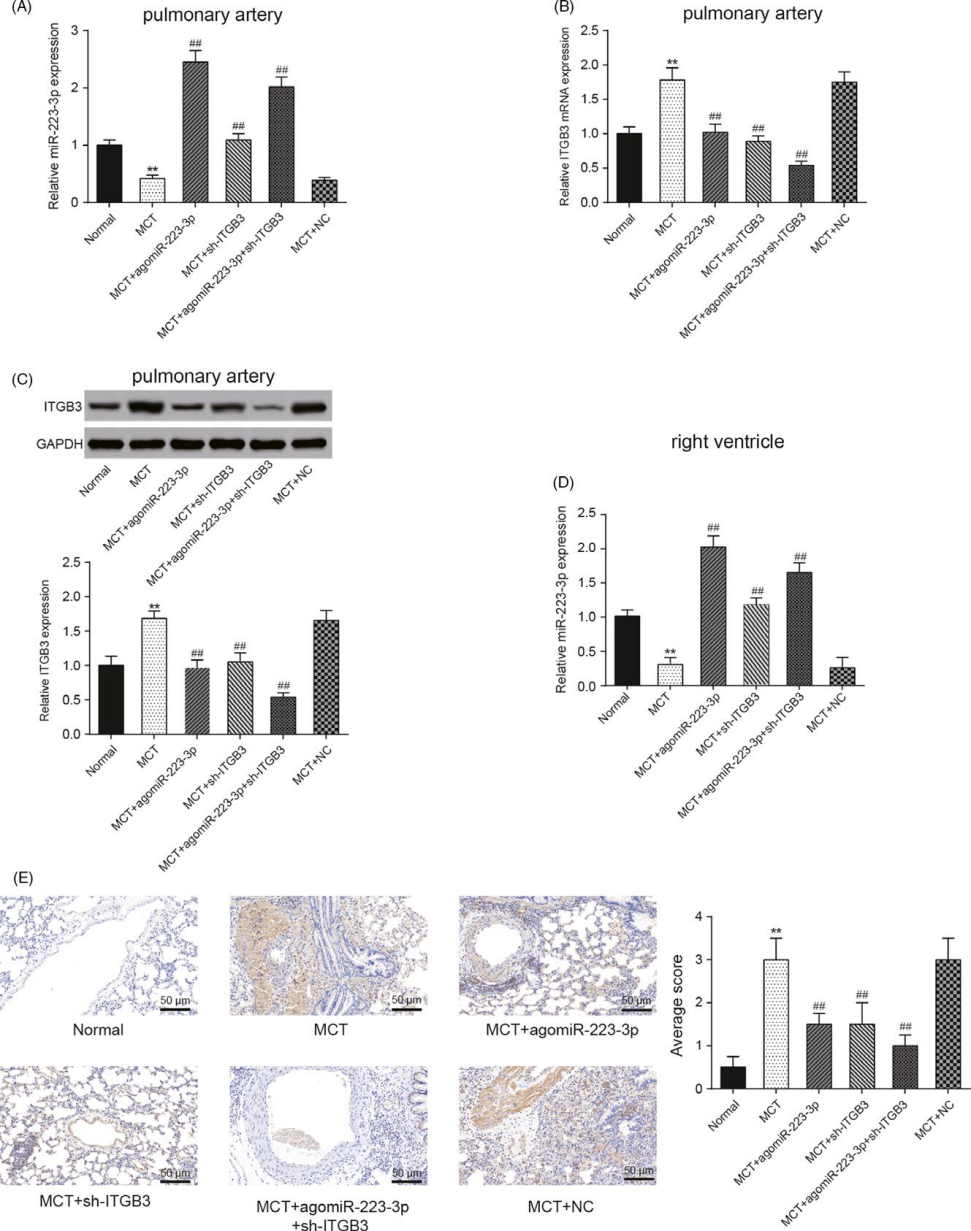
MiR‐223‐3p expression in the pulmonary artery and right ventricle, and *ITGB3* expression in the pulmonary artery. A, MiR‐223‐3p expressions in the pulmonary artery of each group, as measured by qRT‐PCR. B, *ITGB3* expressions in the pulmonary artery of each group, as measured by qRT‐PCR. C, *ITGB3* protein expression in pulmonary artery of each group, as measured by Western blot. D, The miR‐223‐3p expressions of each group in right ventricle. E, IHC analysis of *ITGB3* expressions in each group. Monocrotaline (MCT) + NC: MCT + negative control of MCT + agomiR‐223‐3p and MCT + sh‐ITGB3, n = 8 per group; all experiments were performed in triplicate. Magnification ×200, scale bar: 50 µm, **P < 0.01 compared with the normal group; ^##^ *P *< 0.01 compared with the MCT group

After 4 weeks, pulmonary artery tissues were collected for the extraction of the total RNA and protein for subsequent analyses. After MCT treatment, miR‐223‐3p expression significantly decreased and *ITGB3* mRNA and protein levels increased. Furthermore, treatment with agomiR‐223‐3p resulted in the upregulation of miR‐223‐3p and the suppression of *ITGB3*. In addition, treatment with the sh‐*ITGB3* adenovirus had no effects on miR‐223‐3p expression but hindered *ITGB3* expression. Moreover, co‐treatment with both agomiR‐223‐3p and sh‐*ITGB3* led to the upregulation of miR‐223‐3p expression and the downregulation of *ITGB3* mRNA and protein expression (Figure 14A‐C, *P *< 0.01). Given that Shi et al reported that miR‐223‐3p was downregulated in the lung and right ventricle (RV) due to hypoxia, and the right ventricle is affected by PAH,^35^ we investigated the expression of miR‐223‐3p in the right ventricle of the PAH models. The results were consistent with the results in the pulmonary artery (Figure 14D, *P *< 0.01). Furthermore, immunohistochemical (IHC) analysis demonstrated that *ITGB3* expression in the MCT group was higher compared with the normal group, and moreover, lower *ITGB3* expression was observed in the agomiR‐223‐3p and sh‐*ITGB3* transfection groups. There was an even more obvious decrease in *ITGB3* expression in the group that was co‐treated with agomiR‐223‐3p and sh‐*ITGB3* (Figure 14E, *P *< 0.01).

### 3.10 Pathological features of rat lung tissues and the right ventricular myocardium

**Figure 15:**
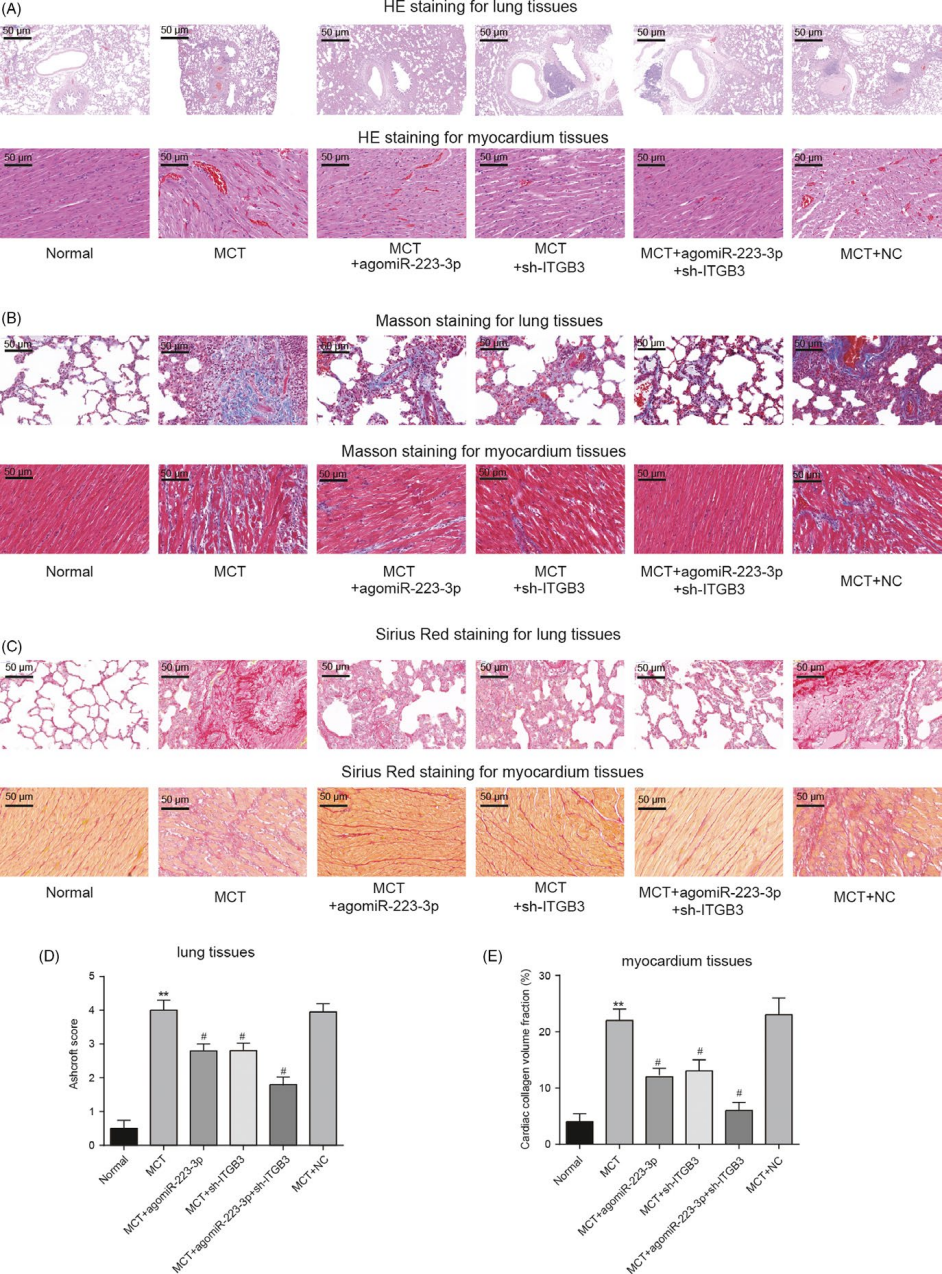
Pathological features of rat lung tissues and right ventricular myocardium. A, Results of HE staining of rat lung tissues and right ventricular myocardium. B, Results of Masson staining of rat lung tissues and right ventricular myocardium. C, Results of Sirius red‐picric acid staining of rat lung tissues and right ventricular myocardium. D, Degree of pulmonary fibrosis was graded and evaluated by the Ashcroft method as described in Materials and Methods. E, Quantitative analysis of collagen volume fraction of myocardium tissues HE: nucleus (blue); cytoplasm (pink); collagen fibres (light pink); erythrocyte (orange). Masson: collagen fibres (blue); muscle fibres (red); nucleus (dark blue); Sirius red‐picric acid: collagen fibres (red); nucleus (green); other yellow, n = 8 per group; all experiments were performed in triplicate. Magnification ×200, scale bar: 50 µm, **P < 0.01 compared with the normal group; ^#^ *P *< 0.05 compared with the MCT group

Pathological staining of rat lung tissues was performed to investigate the effects of miR‐223‐3p and *ITGB3* on rat PAH. First, HE staining revealed severe pulmonary haemorrhage, distinct pulmonary vascular remodelling, severe myocardial tissue haemorrhage and cell morphologic breakage in the MCT group. However, the overexpression of miR‐223‐3p and the silencing of *ITGB3* improved the features (Figure 15A). Moreover, severe fibrosis of lung or myocardial tissues occurred in the MCT group. Both the overexpression of miR‐223‐3p and the silencing of *ITGB3* attenuated this fibrosis to a degree reflected by Masson and Sirius red‐picric acid staining (Figure 15B,C). Quantitative analysis of H&E staining was performed using the Ashcroft scoring method (Figure 15D). The staining of myocardium tissues was quantified (Figure 15E). Both upregulation of miR‐223‐3p and the silencing of *ITGB3* improved this symptom drastically.

## 4 DISCUSSION

Our study demonstrated the roles of miR‐223‐3p and *ITGB3* in PAH. MiR‐223‐3p was downregulated, while *ITGB3* was upregulated in PAH. MiR‐223‐3p attenuated the progression of PAH by suppressing proliferation and decreasing α‐SMA expression in PASMCs, a key cell type constituting the vascular wall of small pulmonary arteries and contributing to pulmonary vascular remodelling.^36^ To ensure that our preclinical findings are consistent with future clinical studies, according to the latest guidelines of optimal methods and more stringent study in PAH^36^ and key principles and guidelines for reporting preclinical research delivered by the National Institutes of Health,^37^ both in vitro and in vivo experiments are important. In our study, the experiments related to PAH were carried out in vivo and in vitro; for example, comprehensive haemodynamics were detected. The results revealed that miR‐223‐3p reduced the levels of mPAP, TRP, EDP and RVSP while increasing the CO of rat models and improving the pathological features caused by MCT. Compared with miR‐223‐3p, *ITGB3* exerted an opposite effect on PAH, remarkably promoting the development of PAH. Moreover, PASMCs were studied in vitro after hypoxia treatment. MiR‐223‐3p inhibited the viability of PASMCs and promoted the apoptosis of PASMCs. In contrast, *ITGB3* promoted the proliferation of PASMCs. Furthermore, our research design was carefully considered, our methodology was well founded, and reasonable interpretation of the data was made to ensure the accuracy of the results.

Previous studies have verified that several miRNAs are differentially expressed and play important roles in PAH. MiR‐206 levels were reported by Jalali et al^38^ to be downregulated in PAH mice. Rhodes et al^39^ reported that miR‐150 levels exhibited the greatest reduction and were associated with survival in patients with PAH. As a significantly downregulated miRNA in PAH, miR‐223‐3p warrants investigation.

The functions of miR‐223‐3p in PAH were revealed in this study, which provided novel insights into the mechanism of PAH; very few studies have reported the effect of miR‐223‐3p. The upregulation of miR‐223‐3p inhibited the viability of PASMCs, promoted the apoptosis of PASMCs and reduced PAH symptoms in rats injected with MCT. Previous research showed that DNA damage can induce the apoptosis of cells.^40^, ^41^ Therefore, we investigated the expression of γ‐H2AX, a DNA damage marker. The results revealed that the expression of γ‐H2AX was increased along with the increase in apoptosis under hypoxic conditions. These results were consistent with the report by Meloche.^9^ MiR‐223‐3p was reported to have an anti‐tumour role in head and neck squamous cell carcinoma and osteosarcoma and suppresses angiogenesis or cell metastasis via targeting cadherin‐6 (CDH6) and other targets.^42^, ^43^ In this study, we proposed that miR‐223‐3p targets *ITGB3* to participate in the process of PAH.

Current researches provide little evidence of the relationship between miR‐223‐3p and *ITGB3* in the context of PAH. Earlier studies reported the targeting relationship between miR‐223‐3p and PARP‐19 ^,^ 44 and IGF‐IR^30^ Therefore, we investigated the expression of PARP‐1 and IGF‐IR Our results showed that the overexpression of miR‐223‐3p suppressed the hypoxia‐induced expression of PARP‐1 and IGF‐IR, which was consistent with earlier research. Furthermore, *ITGB3*, which may be a target of miR‐223‐3p, is known to participate in cell adhesion as well as cell surface‐mediated signalling. Other cell adhesion molecules, such as *CD44*, play critical roles in PAH.^45^ Current studies have verified that *ITGB3* influences cancer cell proliferation and migration.^10^, ^46^, ^47^ Ni et al^10^ verified that upon restoring the expression of *ITGB3*, the effect of miR‐98 on non‐small‐cell lung cancer cell proliferation was partially reversed. Our results support the hypothesis that *ITGB3* promotes the proliferation of PASMCs, and confirm that the knockdown of ITGB3 effectively alleviates PAH progression.

Limitations exist in this study, and further experiments are required to identify the exact mechanism through which miR‐223‐3p and *ITGB3* modulate cell proliferation and migration. MicroRNA‐based therapies will require validation at the clinical level. The extracellular matrix (ECM) reporter interaction is also unclear.

MiR‐223‐3p was downregulated in PAH, while *ITGB3* was upregulated. MiR‐223‐3p attenuated the proliferation of PASMCs and the PAH symptoms in rats induced by MCT, whereas *ITGB3* promoted the deterioration caused by PAH. These data suggested that miR‐223‐3p and *ITGB3* are involved in PAH, and these results uncovered for the first time that miR‐223‐3p inhibits PAH by targeting *ITGB3*. These findings may provide a new therapeutic target for the treatment of PAH.

## ETHICAL APPROVAL

All procedures performed in studies involving animals were in accordance with the ethical standards Beijing Anzhen Hospital.

## CONFLICT OF INTEREST

The authors confirm that there is no conflict of interest.

## AUTHOR CONTRIBUTIONS

Aijun Liu and Yifan Liu critically revised the manuscript; Bin Li and Ming Yang substantially contributed to the conception and design of the work, and drafted the manuscript; Yang Liu and Junwu Su involved in acquisition, analysis and interpretation of the data; Aijun Liu and Yifan Liu revised the manuscript critically, final approval of the version to be published. All authors have read and approved the final article.
